# Emergence of Deviance Detection in Cortical Cultures Through Maturation, Criticality, and Early Experience

**DOI:** 10.1111/ejn.70706

**Published:** 2026-10-07

**Authors:** Zhuo D. Zhang, Amit Yaron, Dai Akita, Tomoyo Isoguchi Shiramatsu, Zenas C. Chao, Hirokazu Takahashi

**Affiliations:** ^1^ Department of Mechano‐Informatics, Graduate School of Information Science and Technology The University of Tokyo Tokyo Japan; ^2^ International Research Center for Neurointelligence (WPI‐IRCN), UTIAS The University of Tokyo Tokyo Japan

**Keywords:** CMOS‐MEA, criticality, deviance detection, dissociated cortical cultures, synaptic plasticity

## Abstract

Mismatch negativity (MMN) in humans is widely interpreted as reflecting deviance detection (DD) within predictive processing frameworks. However, the developmental processes by which DD emerges and matures during early cortical development remain incompletely understood. Here, we longitudinally tracked the development of DD in dissociated cortical cultures grown on high‐density CMOS microelectrode arrays from Day In Vitro (DIV) 10 to DIV30 (Study 1) and compared cultures with and without early structured stimulation at DIV31–35 (Study 2). Cultures were stimulated with oddball and many‐standards control paradigms while spontaneous and evoked activity were recorded. In Study 1, stimulus‐evoked responses at early stages were largely confined to fast components corresponding to direct stimulation effects. From DIV15 onward, robust late responses emerged, and deviant stimuli evoked stronger responses than frequent and control stimuli, marking the emergence of DD as defined in this study. By DIV30, responses became stronger, faster, and more temporally precise. Neuronal avalanche analysis revealed a transition from subcritical to near‐critical dynamics across development. Cultures exhibiting power‐law–like statistics showed larger DD indices, although DD was also observed in non‐critical networks. These findings indicate that critical dynamics are not required for DD, but critical‐like states are associated with more robust and differentiated DD responses as networks mature. In Study 2, both groups shifted toward power‐law–like avalanche distributions, with no significant difference in their developmental trajectories. At DIV31–35, stimulated cultures had earlier peak responses and lower DD indices than controls, but these group differences were not significant. Together, these findings indicate that dissociated cortical cultures can exhibit DD under simplified in vitro conditions and that its developmental trajectory is associated with network maturation and self‐organization toward critical‐like dynamics.

## Graphical abstract

Deviance detection (DD) emerges in dissociated cortical cultures around DIV15 and remains detectable through maturation. Near‐critical network dynamics are associated with stronger DD responses. Early structured stimulation was associated with descriptive differences in response timing and DD strength, although mature‐stage group differences were not statistically significant.

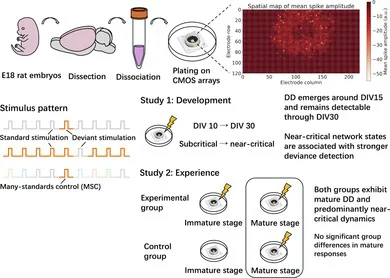

**Abbreviations**
DD: deviance detection
DDI: deviance detection index
DIVs: days in vitro
MMN: mismatch negativity
MMR: mismatch response
MSC: many‐standards control
PSTH: peri‐stimulus time histogram
SSA: stimulus‐specific adaptation
SSAI: stimulus‐specific adaptation index

## 1 Introduction

The ability of neural systems to detect deviations from regular sensory patterns is a well‐documented phenomenon across species. In humans, this function is indexed by the mismatch negativity (MMN), a prominent event‐related potential peaking at ~100–250 ms after the onset of a deviant stimulus (Näätänen et al. 2007). Comparable mismatch responses (MMRs) have been reported in animal models, typically appearing with shorter latencies of 50–150 ms (Javitt et al. 1992; Shiramatsu et al. 2013). Two main processes contribute to these population‐level signals: stimulus‐specific adaptation (SSA) and deviance detection (DD). SSA is a stimulus‐selective reduction in the response to repeated stimuli, with less attenuation of responses to infrequent stimuli. Related adaptation phenomena were described earlier in visual cortex (Movshon and Lennie 1979), while SSA was later established in the auditory oddball literature (Ulanovsky et al. 2003; Taaseh et al. 2011). SSA has been observed across multiple stages of the auditory pathway, including the brainstem, inferior colliculus, thalamus, and auditory cortex (Malmierca et al. 2009; Anderson et al. 2009; Duque et al. 2012, 2018; Anderson and Malmierca 2013; Malmierca et al. 2015). By contrast, DD has been widely studied in cortical circuits as a response pattern in which neurons exhibit enhanced firing to deviant stimuli relative to many‐standards control (MSC) conditions, beyond that attributable to SSA alone (Shiramatsu et al. 2013; Hamm and Yuste 2016; Musall et al. 2017; Parras et al. 2017; Polterovich et al. 2018; Hamm et al. 2021). However, deviance‐related responses have also been reported at subcortical levels, including the inferior colliculus, suggesting that such phenomena are not exclusively cortical (Carbajal and Malmierca 2018). Importantly, DD depends on N‐methyl‐d‐aspartate (NMDA) receptor–mediated synaptic transmission (Shiramatsu et al. 2013; Chen et al. 2015; Zhang et al. 2025).

Developmental studies in humans and animals have shown that MMRs emerge early but undergo substantial refinement with maturation. In newborns, deviant stimuli evoke broad positive slow waves rather than adult‐like negative deflections, indicating an immature form of change sensitivity (Sambeth et al. 2009; Vestergaard et al. 2010). By 2–4 months of age, infants begin to transition from these positive responses to MMN‐like negativities, reflecting the rapid maturation of mechanisms for change detection (Trainor et al. 2003; He et al. 2009). During later childhood, MMN and P3a remain relatively stable, but late discriminative components decline in amplitude, whereas oscillatory activity in the delta and theta bands strengthens in parallel with the development of attentional control (Morales et al. 2023). Animal studies further show that SSA follows a sequential maturation along the auditory pathway—emerging first in the inferior colliculus, then in the thalamus, and finally in the auditory cortex—where cortical maturation is highly experience dependent (Valerio et al. 2024). Together, these findings indicate that deviance‐sensitive responses emerge early but undergo substantial refinement as neural circuits mature, with adaptation‐related processes such as SSA appearing across multiple stages of the auditory pathway. However, the circuit mechanisms through which these responses emerge and develop remain poorly understood.

In addition to developmental changes, deviance‐related responses have been shown to be influenced by sensory experience. Early postnatal noise exposure disrupts the normal maturation of SSA in auditory cortex neurons, whereas the same manipulation in adulthood has little effect (Wang et al. 2019). Conversely, even in mature animals, both passive exposure to specific sounds and the use of a conditioned stimulus in classical conditioning can induce long‐term memory traces and modify MMRs (Kurkela et al. 2016; Shiramatsu and Takahashi 2018, 2021). Consistent with these in vivo findings, in vitro preparations also exhibit experience‐dependent changes in network responses that resemble prediction‐related dynamics. For example, dissociated cortical neurons can learn and classify stimulation patterns through training‐induced structural reorganization (Shao et al. 2025), and organotypic slices trained with temporal sequences can replay learned patterns during spontaneous activity and generate prediction error‐like responses when expected stimuli are omitted (Liu and Buonomano 2025). Together, these findings indicate that sensory experience can shape MMRs in vivo, whereas in vitro studies demonstrate that neuronal networks can develop experience‐dependent response dynamics that resemble predictive processing.

Cultured cortical networks grown on multi‐electrode arrays provide a reductionist model to investigate how neural dynamics and computations emerge from local interactions (Yaron et al. 2025). During in vitro development, dissociated networks self‐organize from sparse, uncoordinated firing into structured activity characterized by synchronous bursts and propagating “network spikes,” reflecting the progressive strengthening of recurrent connectivity (Jimbo et al. 1999; Eytan and Marom 2006; Marom and Shahaf 2002). A hallmark of this process is the emergence of neuronal avalanches whose size distributions approach power laws, suggesting that network activity operates near a critical regime (Beggs and Plenz 2003; Pasquale et al. 2008; Priesemann et al. 2013). Transitions toward criticality depend on excitation–inhibition balance, small‐world topology, and plasticity mechanisms (Massobrio et al. 2015; Yada et al. 2017; Wilting and Priesemann 2019; Stepp et al. 2015; Ikeda et al. 2023, 2025), with cultures often residing in slightly subcritical “reverberating” states (Priesemann et al. 2014; Heiney et al. 2022). Networks approaching criticality have been reported to exhibit enhanced dynamic range, efficient information transmission, and improved learning performance (Kinouchi and Copelli 2006; Haldeman and Beggs 2005; Shew et al. 2009, 2011; Shew and Plenz 2013; Habibollahi et al. 2023). These properties have led to the proposal that near‐critical dynamics may provide a favorable regime for complex network computations.

Building on these insights, high‐density CMOS‐based MEAs enable simultaneous, long‐term recordings from thousands of neurons with subcellular resolution, providing unprecedented access to the spatiotemporal structure of cultured network activity (Berdondini et al. 2005; Müller et al. 2015; Obien et al. 2015; Bakkum et al. 2013, 2019; Yaron et al. 2025; Yada et al. 2017). Recent studies using these platforms have reported predictive‐like response dynamics in cultured neurons. Early stimulus‐evoked responses (0–10 ms) are largely dominated by direct activation, whereas later responses (11–150 ms) reflect synaptic recruitment (Eytan et al. 2003), giving rise to SSA‐ and deviance‐related phenomena analogous to MMRs observed in vivo (Zhang et al. 2025). However, it remains unclear how DD develops over time in vitro and whether its maturation is related to the emergence of critical‐like network dynamics. In this study, we systematically tracked the developmental trajectory of DD in dissociated cortical cultures between DIV10 and DIV30, leveraging high‐density CMOS‐MEA recordings to monitor spontaneous and evoked activity longitudinally. We further tested whether cultures operating closer to criticality exhibit stronger deviance‐sensitive responses and whether early structured stimulation modifies the temporal dynamics of these responses during the mature stage (DIV31–35). This approach allowed us to examine how self‐organized network dynamics relate to the emergence and refinement of deviance‐sensitive responses in simplified cortical circuits.

## 2 Methods

We conducted experiments on dissociated cortical cultures recorded with high‐density CMOS‐MEA. The study consisted of two complementary parts. First, we tracked the developmental trajectories of DD and network dynamics. DD was assessed by repeating oddball and MSC paradigms every 5 days from DIV10 to DIV30, whereas spontaneous network activity was analyzed to quantify neuronal avalanches and characterize network states based on their avalanche size distributions: exponential (subcritical), bimodal (sporadic large avalanches coexisting with smaller ones), or power‐law (near‐critical). Second, we tested the influence of early structured stimulation. One group of cultures received five structured stimulation sessions consisting of the oddball and MSC paradigms during DIV10–30, whereas a control group underwent spontaneous activity recordings without structured stimulation; both groups were then assessed at maturity (DIV31–35) under the same oddball and MSC paradigms.

### 2.1 Preparation of Neuronal Culture

Primary cortical neurons were obtained from Embryonic Day 18 (E18) Wistar rats of both sexes. All procedures were approved by the Animal Experiments Committee of the Graduate School of Information Science and Technology, the University of Tokyo (JA21‐8), and were conducted in accordance with the guidelines of the Japanese Physiological Society.

Pregnant rats were euthanized under deep anesthesia, and cortical tissues were dissected from both hemispheres of E18 fetuses. Following enzymatic digestion with 0.25% trypsin–EDTA (Thermo Fisher Scientific, Waltham, MA, USA), cells were gently dissociated in Neurobasal Plus medium supplemented with B‐27 Plus (Thermo Fisher Scientific) and adjusted to a density of 38,000 cells per 5 μL. A 5‐μL suspension was seeded onto high‐density CMOS microelectrode arrays (MaxOne, MaxWell Biosystems, Zurich, Switzerland) pre‐coated with 0.05% polyethyleneimine (PEI; Sigma‐Aldrich, St. Louis, MO, USA) and laminin (Sigma‐Aldrich, St. Louis, MO, USA). After a 2‐h adhesion period, cultures were covered with 0.6 mL of Neurobasal Plus medium (Figure 1A). Cells were incubated at 36.5°C in a humidified atmosphere containing 5% CO_2_. On the following day, half of the medium was replaced with growth medium consisting of DMEM (Thermo Fisher Scientific), horse serum (Cytiva, Marlborough, MA, USA), GlutaMAX (Thermo Fisher Scientific), and sodium pyruvate (Thermo Fisher Scientific), as described previously (Jimbo et al. 1993; Potter and DeMarse 2001; Yada et al. 2016, 2017; Kubota et al. 2019; Ikeda et al. 2025; Zhang et al. 2025). Thereafter, half‐medium changes were performed every 3–4 days. Recordings were conducted according to the experimental protocols described below.

**FIGURE 1:**
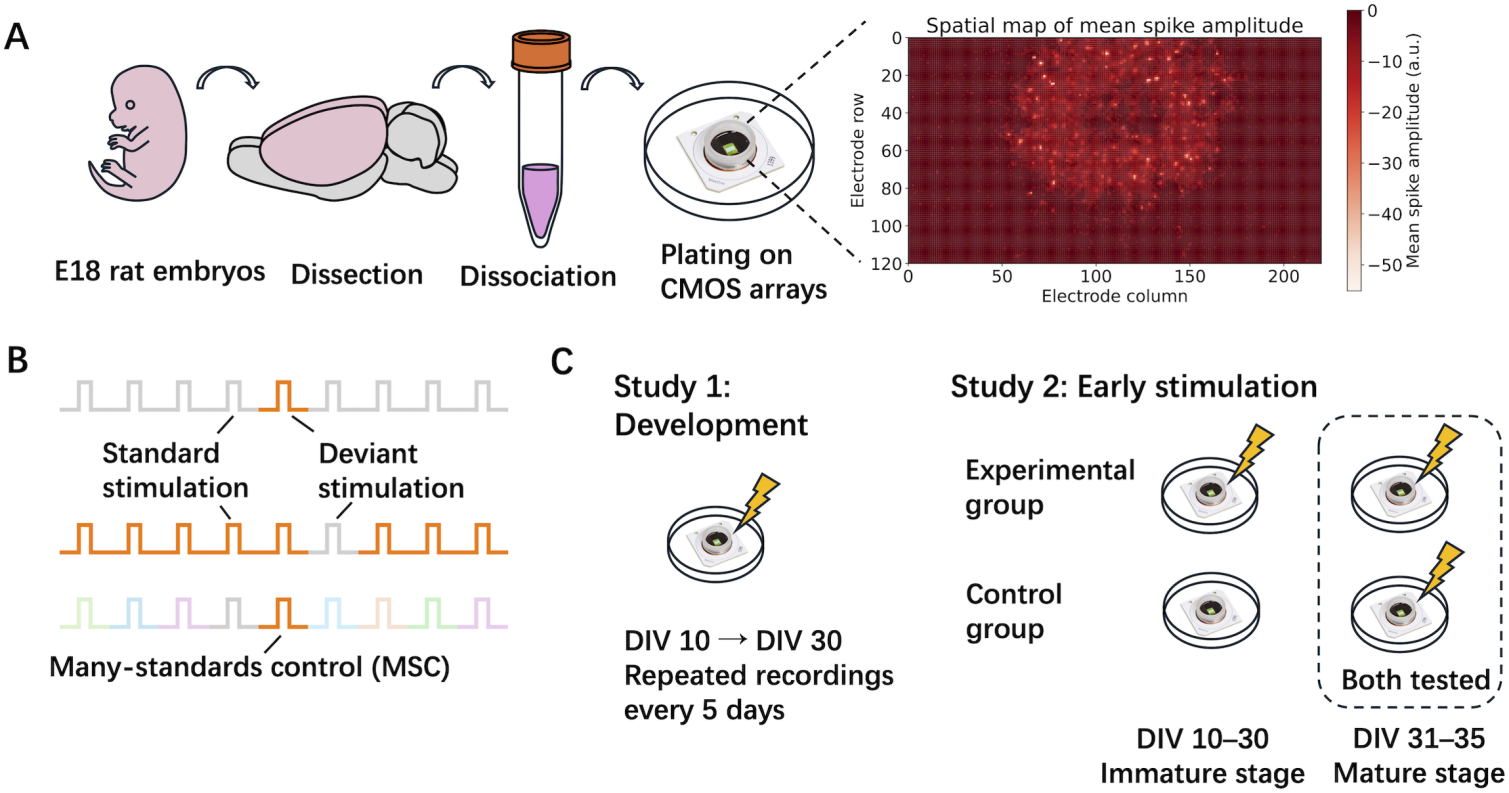
Experimental design and stimulation paradigms. (A) Culture preparation and recording configuration. Cortical tissues were dissected from Embryonic Day 18 (E18) rat embryos, enzymatically and mechanically dissociated, and plated onto high‐density CMOS microelectrode arrays. Neurons attached randomly across the electrode surface. The right panel shows a representative spatial map of mean spike amplitude recorded from the CMOS array, illustrating the heterogeneous distribution of neuronal activity across electrodes (arbitrary units, a.u.). (B) Common stimulation paradigms used in both studies. Oddball and MSC paradigms were applied with a fixed inter‐stimulus interval of 500 ms. In the oddball paradigm, standard stimuli were interleaved with rare deviant stimuli. In the MSC condition, multiple stimulation sites were presented with equal probability. (C) Study 1 (development): The same cultures were recorded at DIV10, 15, 20, 25, and 30. Each session followed the sequence spontaneous recording → flip → rest → flop → rest → MSC → spontaneous recording; rest intervals were 5 min. Study 2 (early stimulation): Cultures were divided into an experimental group and a control group. During DIV10–30, the experimental group underwent spontaneous recording before each of five oddball/MSC sessions, whereas the control group underwent spontaneous recording at the same DIVs without structured stimulation. During daily testing at DIV31–35, both groups were tested using the same oddball and MSC paradigms. Responses were averaged across days within each culture.

A total of *N* = 7 cultures were included in the developmental experiment (Study 1), and *N* = 6 cultures per group were included in the mature‐stage comparison (Study 2). Independent sets of cultures were prepared for each study. Cultures were included if they exhibited stable spontaneous activity and could be reliably recorded throughout the experimental protocol. Cultures that failed to develop stable activity or encountered technical issues (e.g., contamination or recording instability due to hardware‐related issues) were excluded.

### 2.2 Electrophysiological Stimulation and Recording

Electrophysiological experiments were performed using a high‐density CMOS microelectrode array system (MaxOne, MaxWell Biosystems, Switzerland) comprising 26,400 electrodes arranged in a 220 × 120 grid over a 3.85 × 2.1‐mm^2^ area. Each electrode measured 9.3 × 5.45 μm^2^ with an inter‐electrode spacing of 17.5 μm, and up to 1024 electrodes could be simultaneously selected for high‐resolution extracellular recordings. Signals were sampled at 20 kHz.

Because neurons were randomly distributed across the array, spontaneous activity was first assessed by scanning overlapping electrode patches to generate a spatial activity map. Based on this map, 1024 electrodes in highly active regions, preferentially near dense neuronal clusters, were selected for subsequent recordings. This set of recording electrodes remained fixed for each culture across developmental stages (DIV). Spontaneous recordings from these electrodes were used to characterize spontaneous activity, including spike amplitudes and firing‐rate distributions.

Stimulation electrodes were identified from the same activity map. Electrodes located near putative excitatory and inhibitory neurons—classified on the basis of relative spike timing and waveform characteristics—were examined, and sites associated with excitatory activity were chosen for stimulation (Tajima et al. 2017; Ikeda et al. 2025). For each culture, 10 stimulation sites were selected within the recorded region. Each site consisted of six nearby electrodes within the same active cluster, with at least one electrode spacing between electrodes to minimize cross‐activation. Stimulation sites were spatially separated from one another and did not overlap. Once selected, both the recording electrodes and stimulation sites remained fixed across developmental stages within each culture, ensuring that inter‐site distances were stable over time.

Across cultures, electrode selection depended on the spatial distribution of neuronal activity; however, the same selection criteria were applied in all cases, resulting in comparable spatial configurations across cultures and experimental groups. Biphasic voltage pulses (200‐μs positive phase followed by 200‐μs negative phase, 350‐mV amplitude) were delivered to each stimulation site.

### 2.3 Spike Detection

Extracellular signals were band‐pass filtered (300–3000 Hz), and spikes were detected using a threshold‐based method. The detection threshold was set to 5 times the standard deviation of the baseline noise, consistent with established approaches for CMOS‐MEA recordings. Spike times were defined as threshold crossings, and spike sorting was not performed. For visualization and verification, waveform segments around detected events were extracted where applicable. All subsequent analyses were performed using custom‐written scripts in Python.

### 2.4 Experimental Paradigms

To investigate the developmental trajectory of DD in dissociated cortical cultures, two structured stimulation experiments were conducted.

In Study 1, DD was examined across immature to mature developmental stages (DIV10–30). Each culture underwent repeated recording sessions every 5 days (Figure 1C), resulting in five sessions in total. For each session, a 30‐min baseline recording of spontaneous activity was obtained before stimulation, followed by a 30‐min post‐stimulation recording to assess subsequent network dynamics. Stimulation protocols included both the oddball and MSC paradigms, described below (Figure 1B).

In Study 2, we tested whether early‐life exposure to structured stimulation influenced DD at later stages. Cultures were divided into two groups: a stimulated group that received five structured stimulation sessions consisting of the oddball and MSC paradigms during DIV10–30 and a naïve control group that did not receive structured stimulation but underwent spontaneous activity recording at the same developmental time points (Figure 1C). Spontaneous activity was recorded before stimulation, typically for 30 min, although a small number of recordings were approximately 10 min. In the mature phase (DIV31–35), both groups were subjected to the same oddball and MSC paradigms. Evoked responses were summarized across DIV31–35 within each culture. For spontaneous avalanche analyses, all available recordings from this period were used, with missing days omitted and each culture weighted equally; spontaneous recordings were complete in the control group and available for 23 of 30 culture–DIV observations in the experimental group, whereas all oddball/MSC sessions were available. The DIV31–35 interval was treated as the mature assessment period.

Across both experiments, oddball and MSC stimulation paradigms were applied. In the oddball paradigm, three pairs of standard and deviant sites were selected from 10 predefined stimulation locations (Stim A–J; see Section 2.2). Stimuli were presented in a pseudo‐randomized sequence with a 9:1 standard‐to‐deviant ratio. A flip–flop design was used such that standard and deviant roles were reversed across two successive runs. This design was employed to control for site‐specific response biases, as different stimulation sites may exhibit heterogeneous responses due to variations in neuronal distribution and local connectivity. By allowing each site to serve as both standard and deviant, this approach enabled within‐site comparisons under frequent versus rare presentation and minimized spatial confounds. Each oddball session consisted of two successive runs (flip and flop), separated by a 5‐min rest period. This was followed by an additional 5‐min rest before the MSC run. Each run consisted of 600 stimuli delivered at 500‐ms intervals. In the MSC paradigm, all 10 sites (Stim A–J) were presented in a randomized sequence with equal probability (10% each). This condition provided a control for stimulus probability and repetition, allowing deviant responses to be compared with responses to the same stimulation site in the MSC sequence. Each MSC run also consisted of 600 stimuli delivered at 500‐ms inter‐stimulus intervals (ISIs). The stimulus sequence for both paradigms is illustrated in Figure 1B. The combination of oddball and MSC paradigms was chosen to dissociate DD from SSA and to assess whether deviant responses exceed those expected from stimulus probability alone.

### 2.5 Data Preprocessing and DD Analysis

Extracellular signals were band‐pass filtered between 300 and 3000 Hz for spike detection. To minimize stimulation artifacts, the first 1 ms following each stimulus was excluded from the detection window. Visual inspection and prior characterization of stimulus‐evoked responses in cultured networks (Zhang et al. 2025) confirmed that residual electrical artifacts were confined to the first 1 ms following stimulation and did not account for the reproducible early response peaks observed across cultures.

For the oddball paradigm, three stimulation sites (Stim A–C) were selected from the 10 predefined stimulation locations (Stim A–J; see Section 2.2). All pairwise combinations among these three sites were tested using a flip–flop design, resulting in six standard–deviant configurations (A–B, B–A, A–C, C–A, B–C, and C–B). Responses to the candidate sites (A–C) were evaluated in the MSC paradigm, and the site exhibiting the largest evoked response (defined as the total spike count following stimulation) was selected as the test site for subsequent analyses. Importantly, this selection was based solely on overall evoked response magnitude and was independent of differences between standard and deviant conditions. No additional selection experiment or optimization based on DD was performed.

For subsequent analyses, responses were compared for the same stimulation site across conditions; that is, the selected test site was analyzed as standard (frequent), deviant (rare), and within the MSC condition. This within‐site comparison ensured that differences between conditions were not confounded by spatial variability in stimulation responses. Neuronal responses were analyzed under three conditions: standard, deviant, and MSC. All standard stimuli were included in the analysis, without exclusion of standards following deviant stimuli. The selected site was stimulated with the same probability (10%) in the deviant and MSC conditions. The intervening stimuli were delivered to one standard site in the oddball condition and to the nine other sites in the MSC condition.

Stimulus‐evoked firing rates (FRs) were calculated from peri‐stimulus time histograms (PSTHs) constructed with 1‐ms bins. For each culture, PSTHs were first computed by averaging across all recording electrodes (including both active and inactive channels) and across trials for each condition. For experiments involving multiple recording DIVs (e.g., DIV31–35), PSTHs were first computed within each DIV and then averaged across DIVs to obtain a single PSTH per culture. In this study, PSTH refers to the culture‐level response obtained after averaging across electrodes, trials, and (where applicable) DIVs. Population‐averaged PSTH refers to the average of these culture‐level PSTHs across cultures.

SSA was quantified using an SSA index (SSAI):

$$\text{SSAI}\left(R\right)=\frac{R-R_{\mathrm{std}}}{R+R_{\mathrm{std}}},$$

where R represents the evoked response to deviant ($R_{\mathrm{dev}}$) or MSC ($R_{\mathrm{MSC}}$) stimuli and $R_{\mathrm{std}}$ is the response to standard stimuli. Here, the response (*R*) was defined as the mean value of the culture‐level PSTH within the late post‐stimulus window. For developmental analyses (DIV10–30), the late window was defined as 11–200 ms to capture prolonged late responses observed at early stages, whereas for mature‐stage analyses (DIV31–35), a window of 11–150 ms was used, reflecting the more temporally confined responses at later stages. These windows exclude the initial 10 ms dominated by early responses and primarily reflect synaptic transmission–driven network activity (Zhang et al. 2025). A positive SSAI indicates that responses to deviant or MSC stimuli exceeded those to standards, whereas a greater SSAI for deviant compared with MSC indicates a larger response to deviant than to MSC stimuli. For the time‐resolved analysis, the instantaneous DEV–STD or MSC–STD difference was normalized by the sum of the corresponding mean responses over 0–500 ms, rather than by the instantaneous sum used in the conventional SSAI formulation.

To directly compare deviant and MSC responses, we further defined a deviance detection index (DDI):

$$\mathrm{DDI}=\frac{R_{\mathrm{dev}}-R_{\mathrm{MSC}}}{R_{\mathrm{dev}}+R_{\mathrm{MSC}}}.$$

A positive DDI indicates stronger responses to deviant than to MSC stimuli, providing the operational measure of DD used here.

To further assess cross‐site overlap, responses were compared for the selected test site across standard, DEV, and MSC conditions. Responsive recording channels were identified independently of the MSC‐based test‐site selection, separately for the early (2–10 ms) and late (11–150 ms) windows. DEV and MSC responses contributed equally to the response and baseline statistics, and a channel was considered responsive when the balanced post‐stimulus response exceeded the corresponding baseline mean by more than 2 SD. Baseline activity was estimated from the prestimulus interval (−50 to 0 ms). For the late window, the full 50‐ms prestimulus interval was used, whereas for the early window, six non‐overlapping 8‐ms segments from −48 to 0 ms were used to match the response‐window duration. Responses to the other MSC sites were evaluated using the same matched‐baseline criterion. For each culture, the two oddball contexts involving the selected test site were averaged equally. We then quantified the fraction of responsive channels also activated by the oddball standard or by other MSC sites and defined overlap load as the number of other MSC sites activating each channel (0–9).

In addition to SSAI and DDI, we also quantified temporal features of the evoked responses. Response duration was defined as the time from stimulus onset to the end of the longest contiguous post‐stimulus response segment exceeding a threshold of three standard deviations above baseline activity. The peak latency was defined as the time from stimulus onset to the maximum of the culture‐level PSTH within the late response window (11–150 ms).

Statistical inference was performed at the culture level unless otherwise specified. Each data point in culture‐level comparisons represents one independent culture. For developmental analyses (DIV10–30), *N* = 7 cultures were included. For mature‐stage comparisons (DIV31–35), *N* = 6 cultures were analyzed in each group (experimental vs. control).

### 2.6 Avalanche Definition

Neuronal avalanches were defined as spatiotemporal clusters of spikes identified through temporal contiguity. All spikes detected across electrodes were merged into a single time‐ordered sequence (“array‐wide” spike train). If the interval between two consecutive spikes was shorter than a threshold duration ∆t, those spikes were assigned to the same avalanche. If no subsequent spike occurred within ∆t, the preceding spike was considered an avalanche of size one (single‐spike event). In this way, each spike belonged to exactly one avalanche, and avalanches are separated by silent periods larger than ∆t. The threshold ∆t was determined according to the intrinsic activity timescale: On each recording day, ∆t was set to the mean inter‐spike interval of the network, computed from the array‐wide spike train by pooling spikes across all active electrodes. The method follows established approaches (Pasquale et al. 2008; Tetzlaff et al. 2010; Yada et al. 2017) and does not rely on a fixed bin size, thereby avoiding bias introduced by arbitrary temporal discretization.

### 2.7 Characterization of Avalanche Distributions

For each recording, we analyzed the probability distribution of avalanche sizes *s* (number of spikes per avalanche). Two candidate models were fitted: a power‐law distribution and an exponential distribution (Yada et al. 2017). The probability mass functions were defined as follows:

$$P_{\alpha }\;\left(s\right)=\left\{\begin{array}{c} cs^{-\alpha },s_{\min}\leq s\leq s_{\max}\;\\ 0,\text{otherwise} \end{array},\right.$$

$$P_{\lambda }\left(s\right)=\left\{\begin{array}{c} ce^{-\mathrm{\lambda s}},s_{\min}\leq s\leq s_{\max}\;\\ 0,\text{otherwise} \end{array},\right.$$

where c is a normalization constant.

The fitting range of the power‐law was defined from the minimum cutoff $s_{\min}=2$ (excluding single‐spike avalanches) up to the maximum avalanche size observed ($s_{\max}$). The exponential distribution was fitted across the entire size range. Because very small avalanches (particularly size 1) often deviate from ideal scaling, power‐law fits were restricted to avalanches with s≥2 ($s_{\min}=2$).

The power‐law exponent α and exponential rate parameter λ were estimated using maximum likelihood estimation (MLE). For a dataset of avalanche sizes ${\left\{s_{i}\right\}}_{i=1}^{N}$, the log‐likelihood functions were as follows:

$$\log L_{\mathrm{pl}}\left(\alpha \right)=\sum_{i=1}^{N}\log P_{\alpha }\left(s_{i}\right),$$

$$\log L_{\mathrm{ep}}\left(\lambda \right)=\sum_{i=1}^{N}\log P_{\lambda }\left(s_{i}\right).$$

Here, α denotes the scaling exponent of the power‐law distribution, and λ denotes the decay rate of the exponential distribution. For clarity, we refer to the corresponding model probabilities as $P_{\alpha }$ (power‐law) and $P_{\lambda }$ (exponential).

Goodness‐of‐fit was assessed using the Kolmogorov–Smirnov (KS) statistic:

$$D=\max_{s}\left|F_{\mathrm{emp}}\left(s\right)-F_{\text{model}}\left(s\right)\right|,$$

where *F*(*s*) denotes the cumulative distribution function (CDF). A lower KS statistic indicates a closer fit. To compare relative model support, we calculated the log‐likelihood ratio (LLR):

$$\mathrm{LLR}=\log L_{\mathrm{pl}}-\log L_{\mathrm{ep}},$$

and the Akaike information criterion (AIC):

AIC=−2logL+2k,

where k=1 is the number of free parameters. The model with the lower AIC was considered better supported, unless $\left|∆\mathrm{AIC}\right|<2$, in which case the models were regarded as indistinguishable.

We further note that KS‐based goodness‐of‐fit measures are less sensitive to deviations in the tail of the distribution. Therefore, model selection and classification were interpreted in conjunction with overall distributional shape, rather than relying solely on goodness‐of‐fit statistics.

### 2.8 Classification Criteria

Avalanche size distributions were classified into exponential, bimodal, or power‐law types, following previous work (Yada et al. 2017). We note that this classification is operational and intended to capture differences in overall distributional regimes rather than to provide a strict identification of underlying statistical models.

First, bimodality was assessed by examining the largest gap in avalanche sizes on a logarithmic scale. The maximum difference between consecutive values in $\log_{10}\left(s\right)$ was defined as the gap value G. If G exceeded the threshold defined as the third quartile (Q3) of all gap values across the dataset, the distribution was labeled as bimodal, reflecting the presence of unusually large avalanches separated from the main body of smaller events.

For non‐bimodal distributions, we compared the fits of power‐law and exponential models.

- A distribution was classified as power‐law if the LLR favored the power‐law fit (LLR>0) and the KS statistic for the power‐law fit was below the acceptance threshold.
- Conversely, a distribution was classified as exponential if LLR<0 and the exponential fit satisfied the KS criterion.

When both models provided comparable fits, additional criteria were applied. Specifically, when the difference in *AIC* (ΔAIC) was smaller than the standard cutoff (∣ΔAIC∣<2), the ratio of KS statistics (acceptance tolerance) was used to resolve the classification, assigning the distribution to either power‐law or exponential. Using this combination of gap‐based and statistical criteria (KS, LLR, ΔAIC), each avalanche distribution was ultimately classified as power‐law, exponential, or bimodal.

### 2.9 Statistical Analysis

All statistical analyses were performed in Python and are reported as mean ± SEM unless otherwise specified. Normality was assessed using the Shapiro–Wilk test where appropriate. SSAI values for deviant and MSC conditions were each tested against zero using two‐tailed Wilcoxon signed‐rank tests. Direct comparisons between deviant and MSC responses were performed using two‐tailed paired Wilcoxon signed‐rank tests. DDI values were tested against zero using Wilcoxon signed‐rank tests; for the mature‐stage analysis (DIV31–35), a one‐sided test with the alternative hypothesis DDI > 0 was used, consistent with the directional definition of DD as DEV > MSC.

Developmental changes in evoked FR (DIV10–30; 0–500 ms after stimulation) were analyzed with a linear mixed‐effects model. Fixed effects were DIV, stimulus condition (standard, deviant, and MSC), and their interaction. Random intercepts were included for culture and culture–DIV recording. Fixed effects were evaluated with Wald *χ* ^2^ tests. Pairwise condition contrasts were adjusted using the Holm procedure across all 15 comparisons, comprising three contrasts at each of the five DIVs.

Recording‐level distributions of DDI and FR across avalanche categories were summarized descriptively, with Cliff's delta (*δ*) reported as an effect size where applicable. The association between DDI and FR was summarized using Spearman's *ρ*. Inferential testing of DDI across avalanche categories was performed using a mixed‐effects model with avalanche category and DIV as fixed effects and culture as a random intercept, with pairwise category contrasts adjusted using the Holm procedure.

For the cross‐site overlap analysis, fractions of overlapping channels were compared with two‐sided Wilcoxon signed‐rank tests across cultures. We estimated a slope for the DEV–MSC response difference against MSC overlap load in each culture and then tested for a positive slope using a one‐sided exact sign‐flip test.

For mature‐stage group comparisons (DIV31–35), measurements were first averaged across days within each culture, leaving six independent cultures per group. DDI, deviant‐response peak latency, DEV‐evoked FR, and deviant‐response duration were compared using two‐sided Mann–Whitney *U* tests, with Benjamini–Hochberg false discovery rate (FDR) correction across the four measures. For the multivariate comparison, the four culture‐level features were *z*‐standardized before Euclidean distances were calculated. PERMANOVA significance was evaluated using all 924 possible group‐label assignments.

As a sensitivity analysis, all available DIV31–35 recordings were retained in linear mixed‐effects models. Each model included group, centered DIV, and their interaction as fixed effects and culture as a random intercept. FDR correction was applied across the four response measures.

Group differences in developmental avalanche‐category trajectories were assessed using culture‐level exact permutation tests based on the sum of DIV‐specific Pearson contingency‐table statistics, with group labels permuted while preserving each culture's longitudinal trajectory. Only cultures with complete DIV10–30 trajectories were included. For mature‐stage analyses, category proportions were calculated within each culture from available DIV31–35 recordings, with missing days omitted and cultures weighted equally; overall category composition was compared using the summed squared difference between group mean proportions and power‐law proportion using the absolute difference between group means.

ChatGPT and Codex (OpenAI) were used to assist with language editing and consistency checking during manuscript revision. All AI‐assisted content was reviewed and verified by the authors, who take full responsibility for the final manuscript.

## 3 Results

### 3.1 Developmental Emergence of DD

#### 3.1.1 Emergence and Persistence of DD Across Development

We first investigated how DD emerges and develops in vitro by analyzing neuronal responses to standard, deviant, and MSC stimuli across five developmental stages (DIV10, 15, 20, 25, and 30). All statistical comparisons reported below were performed at the level of independent cultures (*N* = 7 per developmental stage).

At DIV10, dissociated cortical cultures already exhibited spontaneous network activity, including synchronous bursts (Figure 2A,B). These raster plots illustrate representative 5‐min segments of spontaneous activity from cultures that were later classified as exhibiting exponential, bimodal, or power‐law avalanche size distributions based on analyses of the full spontaneous recordings (see Figure 6). However, stimulus‐evoked responses at this stage were largely restricted to early components, reflecting direct electrical activation (Figure 3A). Clear late responses—commonly attributed to synaptically mediated network recruitment (Jimbo et al. 2000; Eytan and Marom 2006; Bakkum et al. 2008; Kermany et al. 2010; Gal et al. 2010; Dranias et al. 2013; Zhang et al. 2025)—were absent. From DIV15 onward, stimulation consistently evoked late responses whose amplitudes showed a general increase across developmental stages (Figure 3B–E). Evoked responses were analyzed within a 0–500‐ms window following stimulus onset. Given the fixed ISI of 500 ms, this window also includes activity immediately preceding the next stimulus.

**FIGURE 2:**
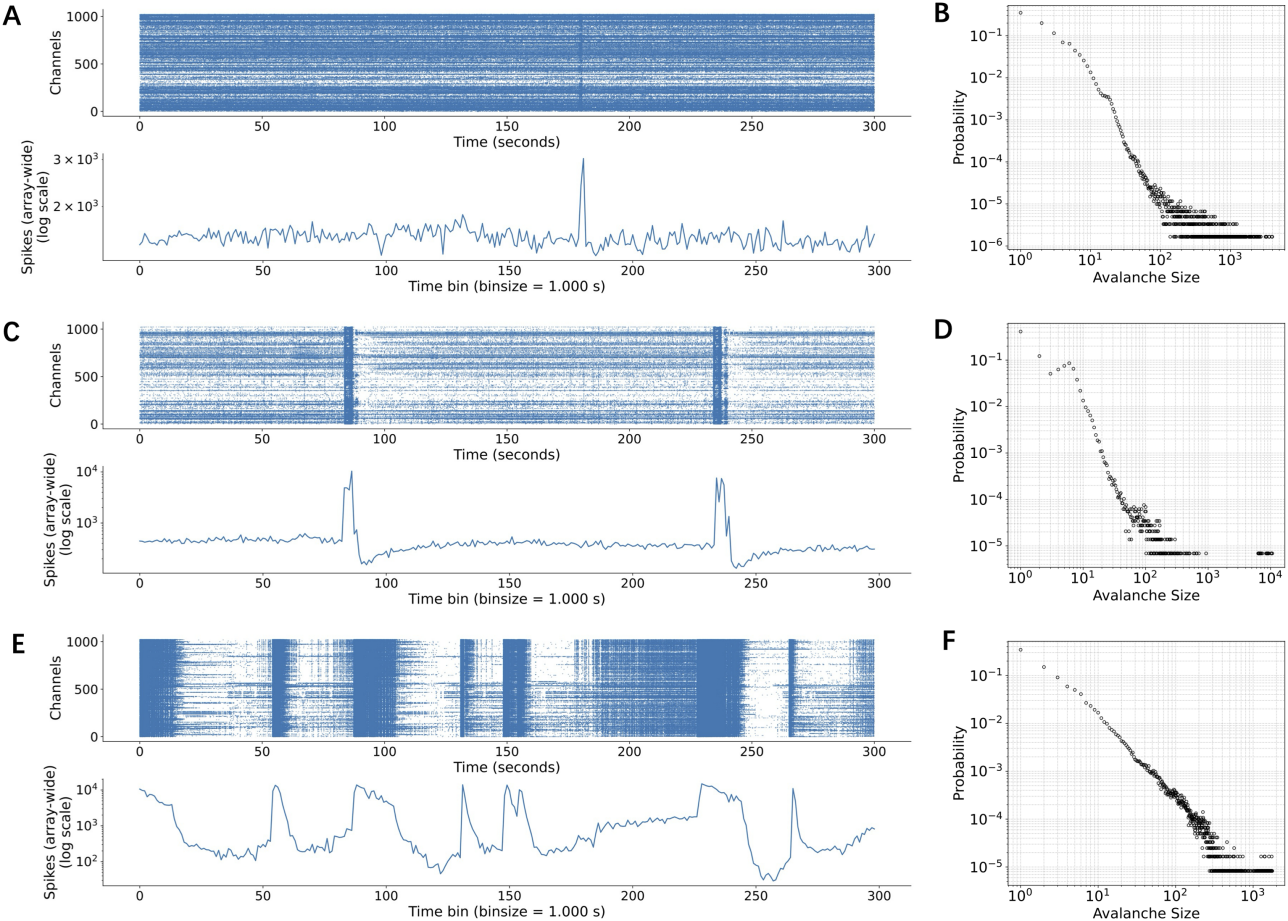
Representative spontaneous activity and corresponding avalanche size distributions across avalanche categories. (A, B) Example culture classified as exponential at DIV10. (A) A representative 5‐min segment of spontaneous activity, shown as a raster plot of spikes recorded from 1024 channels (upper) and the corresponding array‐wide spike counts over time (lower). (B) Avalanche size distribution computed from the full 30‐min spontaneous recording of the same culture. (C, D) Example culture classified as bimodal at DIV10. (C) Representative 5‐min spontaneous activity segment displayed as in (A). (D) Avalanche size distribution computed from the full 30‐min spontaneous recording of the same culture. (E, F) Example culture classified as power‐law at DIV30. (E) Representative 5‐min spontaneous activity segment displayed as in (A). (F) Avalanche size distribution computed from the full 30‐min spontaneous recording of the same culture. Avalanches were defined as contiguous periods of population activity separated by silent intervals (see Section 2). Classification into avalanche categories was based on the full avalanche size distributions (see Figure 6).

**FIGURE 3:**
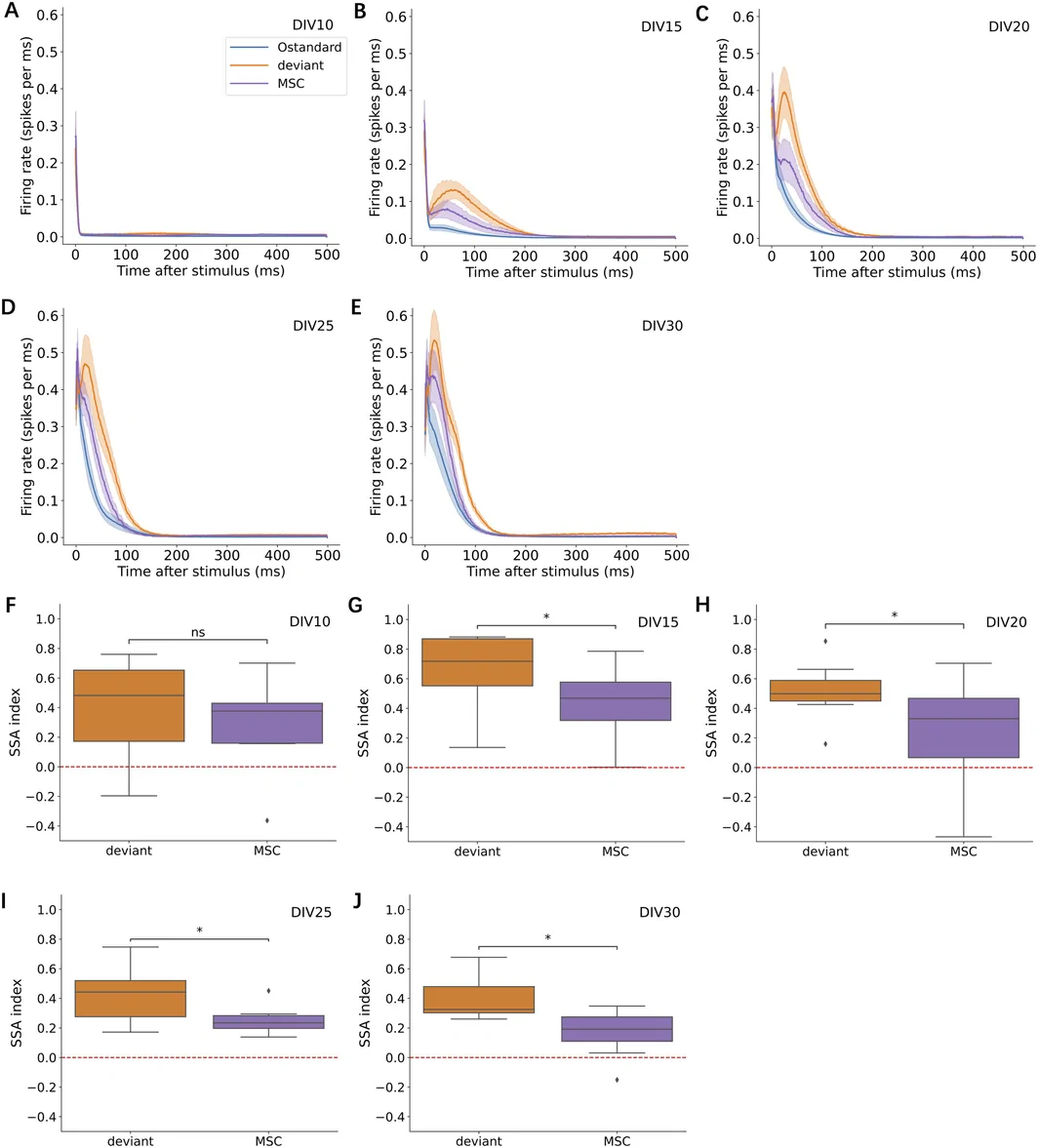
Developmental changes in deviance detection. (A–E) Population‐averaged peri‐stimulus time histograms (PSTHs) at different developmental stages: (A) DIV10, (B) DIV15, (C) DIV20, (D) DIV25, and (E) DIV30. PSTHs were obtained by averaging across all recording channels within each culture and then across cultures. Shaded regions indicate ±SEM across cultures (*n* = 7). Late responses (11–200 ms) emerged from DIV15 onward and increased in amplitude with maturation. (F–J) Stimulus‐specific adaptation indices (SSAIs) for deviant and MSC responses across developmental stages: (F) DIV10, (G) DIV15, (H) DIV20, (I) DIV25, and (J) DIV30. Each box plot shows data from *n* = 7 independent cultures. Horizontal lines indicate medians, boxes indicate interquartile ranges, and whiskers denote the full data range. *Statistical comparisons were performed using two‐tailed Wilcoxon signed‐rank tests: *p* < 0.05 (see Section 3 for details).

Importantly, spatial maps of stimulus‐evoked activity revealed a clear temporal dissociation. Early responses (0–10 ms) remained spatially confined to the vicinity of the stimulated electrode, whereas late responses (11–150 ms) were characterized by widespread recruitment across the network (Zhang et al. 2025). During this late phase, activity patterns evoked by standard, deviant, and MSC stimulation largely overlapped at the population level.

For the developmental analyses, a broader late response window was used. In mature cultures, the late response window has typically been defined as 11–150 ms (Zhang et al. 2025), whereas at earlier developmental stages, particularly DIV15–20, evoked responses often extended beyond 150 ms (Table 1). We therefore used an expanded 11–200‐ms late response window for the calculation of SSAI and DDI in the developmental analyses.

**TABLE 1:** Response duration across developmental stages. Response duration (ms, mean ± SD) for standard (STD), deviant (DEV), and many‐standards control (MSC) stimuli at DIV15, 20, 25, and 30. Values were computed at the level of independent cultures (*N* = 7 cultures per developmental stage).

Days in vitro	STD	DEV	MSC
DIV15 (ms)	225.0 ± 72.4	244.7 ± 48.5	174.3 ± 78.1
DIV20 (ms)	198.6 ± 46.4	209.4 ± 31.0	147.6 ± 47.0
DIV25 (ms)	162.7 ± 50.3	146.0 ± 32.0	125.4 ± 29.3
DIV30 (ms)	160.4 ± 41.8	138.1 ± 13.4	129.3 ± 27.3

With respect to SSA, the SSAI for deviant responses became significantly greater than zero from DIV15 onward, indicating stronger late responses to deviants compared with standards (*p* = 0.078 at DIV10, *p* < 0.05 at later stages; one‐sample Wilcoxon signed‐rank test, two‐tailed). Deviant responses also exhibited significantly higher SSAI values than MSC responses from DIV15 onward (*p* = 0.218 at DIV10, *p* < 0.05 at later stages; Wilcoxon signed‐rank test; Figure 3F–J), indicating the emergence of DD at this stage. SSAI values for MSC stimuli were not significantly different from zero at DIV10 and DIV20 (*p* = 0.078 and *p* = 0.218, respectively) but became significant at DIV15, DIV25, and DIV30 (all *p* < 0.05; Wilcoxon signed‐rank test). Analyses of the DDI further showed that values were not significantly above zero at DIV10 (*p* = 0.078) but became significant from DIV15 onward (DIV15: *p* = 0.0234; DIV20: *p* = 0.0078; DIV25: *p* = 0.0234; DIV30: *p* = 0.0156; Figure 4A). Although small non‐zero DDI values were obtained at DIV10, these likely reflect weak fluctuations within the 11–200‐ms window rather than DD, as no robust late responses were present at this stage (Figure 3A). These findings suggest that deviance sensitivity emerges around DIV15 and becomes more robust and consistent with further maturation.

**FIGURE 4:**
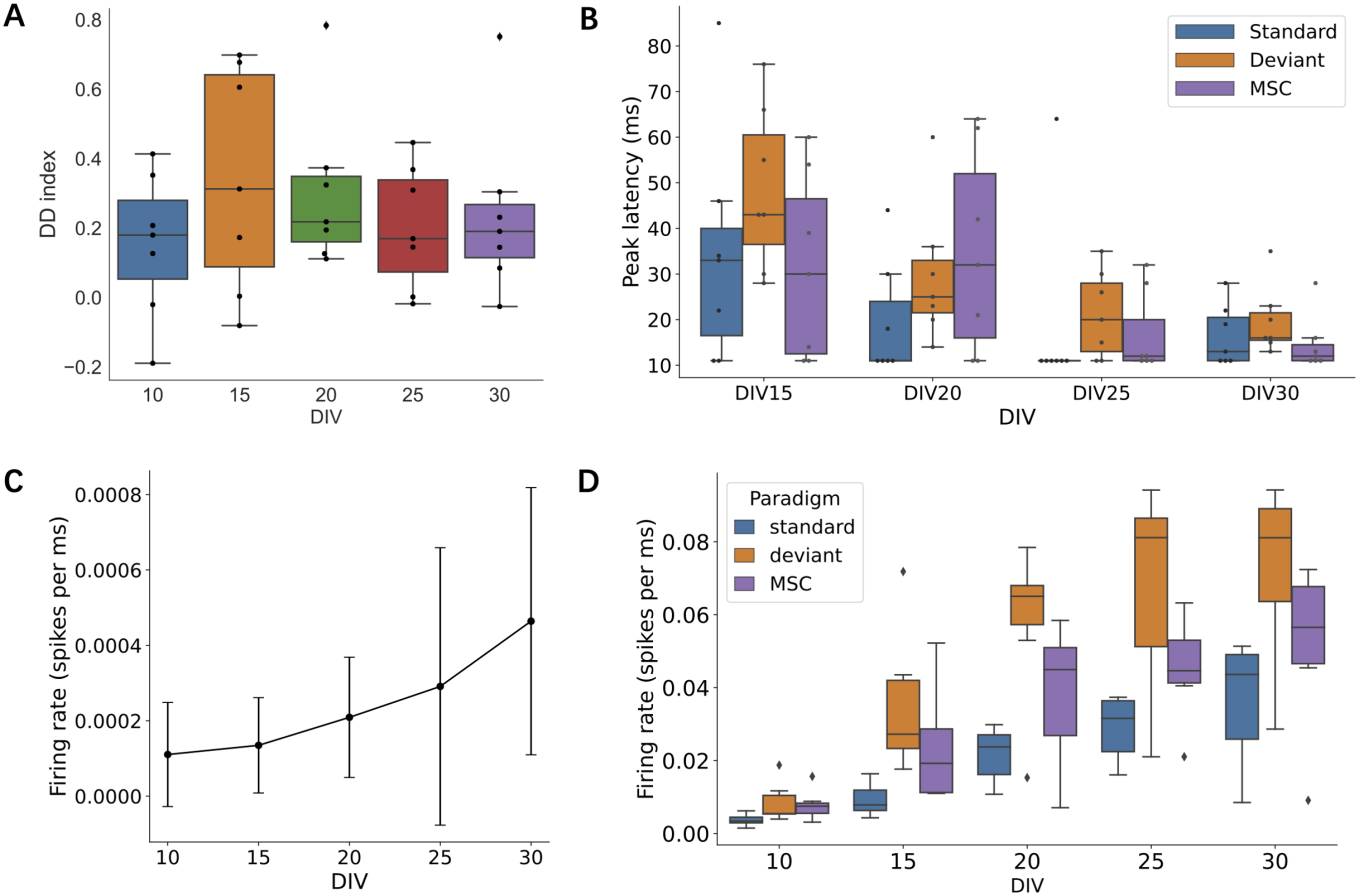
Developmental trajectory of deviance detection and network activity. (A) Deviance detection index (DDI) across developmental stages (DIV10–30), computed from late responses (11–200 ms). (B) Peak latencies of standard, deviant, and MSC responses from DIV15 to DIV30, defined as the time to the maximum of the culture‐level PSTH within the late response window (11–150 ms). (C) Spontaneous firing rates measured during baseline recordings. (D) Mean evoked firing rates, defined as the average value of the culture‐level PSTH within the 0–500‐ms post‐stimulus window. Each data point represents one independent culture (*n* = 7 per developmental stage). Statistical analyses corresponding to these data are described in Section 3.

In parallel, peak latencies of deviant responses shifted toward earlier time points between DIV15 and DIV30 (Figure 4B), consistent with a gradual acceleration of evoked network responses during maturation. Peak latency distributions for standard and MSC responses showed a similar overall tendency, with shorter latencies generally observed at later developmental stages (DIV25–30) than at earlier stages (DIV15–20; Figure 4B). However, in some recordings, particularly when late responses were weak, no distinct secondary peak was observable within the predefined late response window (11–150 ms). In these cases, latency estimates were influenced by the lower boundary of the analysis window. To provide a more robust characterization of temporal response refinement across development, response duration was additionally quantified. Response duration decreased consistently with maturation across stimulus conditions (Table 1), indicating improved temporal precision of network responses. Specifically, shorter response durations reflect a more temporally confined activation of the network following stimulation, suggesting reduced temporal dispersion of spiking activity and more precise timing of population responses.

Spontaneous FRs (Figure 4C) and mean evoked FRs (Figure 4D) increased across developmental stages, indicating a general maturation of network excitability and stimulus responsiveness. Evoked response amplitude was defined as the average value of the culture‐level PSTH within the post‐stimulus window. In contrast, spontaneous FRs were computed from baseline recordings in the absence of stimulation. To formally assess developmental and stimulus‐related effects while accounting for repeated measurements, we used the mixed‐effects model described in Section 2.9. The model detected effects of DIV (Wald *χ* ^2^(4) = 115.776, *p* = 4.26 × 10^−24^), stimulus condition (Wald *χ* ^2^(2) = 190.319, *p* = 4.71 × 10^−42^), and their interaction (Wald *χ* ^2^(8) = 38.260, *p* = 6.74 × 10^−6^). Holm‐corrected DEV–MSC contrasts were not significant at DIV10 (*p* = 0.8866) but showed larger deviant responses at DIV15 (*p* = 0.0350), DIV20 (*p* = 0.0001001), DIV25 (*p* = 0.0000216), and DIV30 (*p* = 0.0001001).

Together, these findings indicate that as dissociated cortical networks develop from DIV10 to DIV30, stimulus‐evoked responses become stronger, faster, and more temporally precise. Differentiation among stimulus conditions also emerged across development: DD responses were not reliably detectable at DIV10 but became observable from approximately DIV15 onward and persisted at later stages, whereas overall response dynamics continued to refine.

We next asked whether the larger deviant response could reflect greater cross‐site adaptation in the MSC condition. The same test site was compared across conditions, but other MSC sites could recruit some of the recording channels that responded to it. Such shared activation could reduce the subsequent MSC response to the test site. We therefore quantified cross‐site response overlap (Figure 5; see Section 2.5 for channel selection).

**FIGURE 5:**
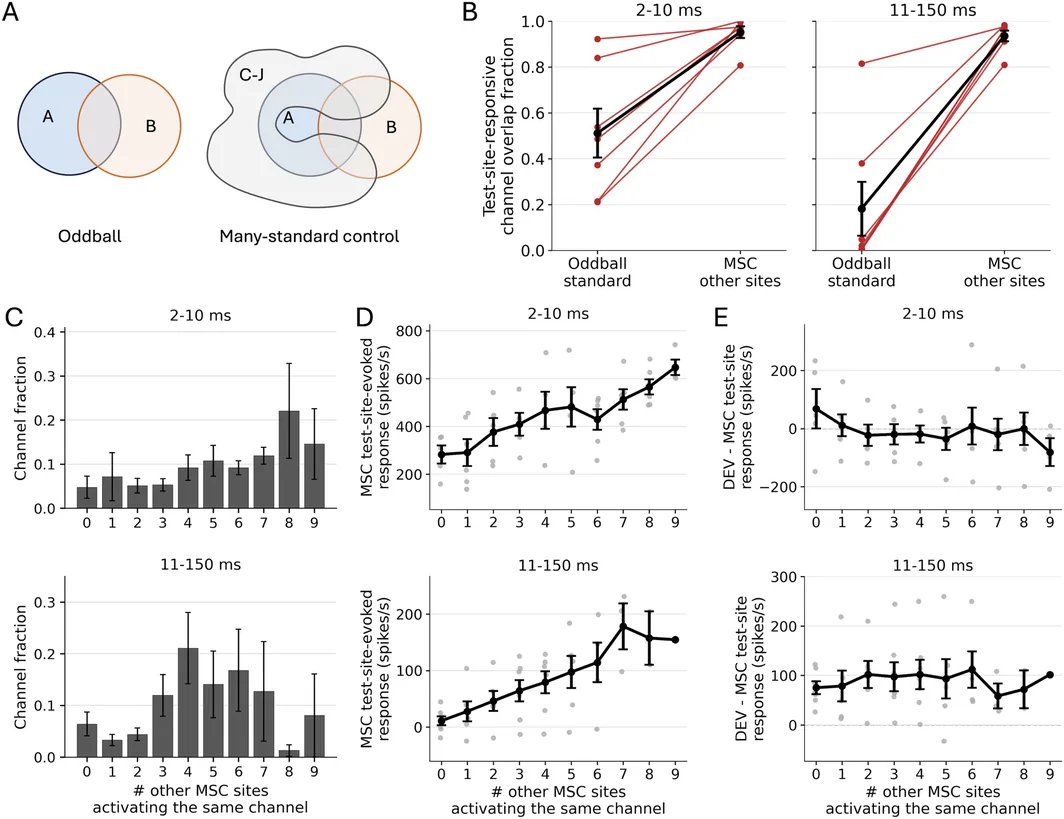
Cross‐site response overlap and the DEV–MSC contrast. (A) Adaptation hypothesis: Other stimulation sites may activate recording channels that also respond to the test site, reducing its subsequent response. The test site is the same stimulation site used in standard, deviant, and MSC conditions. (B) Among recording channels that responded to stimulation at the test site, the fraction also activated by the oddball standard site or by at least one of the nine other MSC stimulation sites. Channels were selected separately for each window using equally weighted DEV and MSC responses (Section 2.5). (C) Distribution of MSC overlap load: The number of other MSC sites activating each responsive channel (0–9). (D) MSC test‐site response as a function of overlap load. (E) DEV–MSC response difference as a function of overlap load. Responses are shown for the early (2–10 ms) and late (11–150 ms) windows. Data are mean ± SEM across cultures where applicable; statistical results are reported in Section 3.1.1.

Among recording channels that responded to the test site, overlap with other MSC stimulation sites was greater than overlap with the oddball standard site. The respective fractions for the oddball standard and other MSC sites were 51.2% ± 10.6% and 95.3% ± 2.5% in the early window (2–10 ms; two‐sided Wilcoxon signed‐rank test, *p* = 0.0156) and 18.2% ± 11.7% and 93.6% ± 2.3% in the late window (11–150 ms; *p* = 0.0156; Figure 5B). Thus, the additional MSC sites did recruit many of the same channels as the test site.

MSC responses were larger in recording channels with higher overlap loads (Figure 5D). We then tested whether the DEV–MSC difference increased with the number of other MSC stimulation sites that evoked a detectable response in each recording channel (MSC overlap load; Figure 5C–E). Slopes of the DEV–MSC difference against overlap load were not significantly positive across cultures: −4.97 ± 5.44 spikes/s per additional site in the early window (one‐sided exact sign‐flip test, *p* = 0.8125) and 1.11 ± 4.29 spikes/s per additional site in the late window (*p* = 0.4141; Figure 5E). Defining responsive channels from DEV alone or MSC alone gave the same qualitative result.

We next examined recording channels with zero overlap load: No other MSC stimulation site evoked a detectable response in these channels. In the late window, their DEV–MSC difference was positive in all seven cultures (75.3 ± 13.2 spikes/s; two‐sided Wilcoxon signed‐rank test against zero, *p* = 0.0156). This finding argues against measured cross‐site overlap as the sole explanation for the late DEV > MSC effect. Late‐window zero‐overlap channels were present in all seven cultures, with 7–24 channels per oddball context; all 14 context‐level zero‐overlap bins contained at least three channels.

#### 3.1.2 Relationship Between Network Criticality Stage and DD

We next investigated whether and how the development of DD is associated with the maturation of network dynamics. As dissociated cortical networks mature, their spontaneous activity shows a shift from predominantly subcritical toward more critical‐like dynamics (Tetzlaff et al. 2010; Yada et al. 2017). This transition may reflect ongoing synaptic wiring and rewiring, during which GABA‐mediated giant depolarizing potentials facilitate the integration of immature neurons (Sipila 2005; Le Magueresse and Monyer 2013). Building on these observations, we therefore hypothesized that the criticality state of the network may modulate the emergence of DD. To test this, we examined the relationship between avalanche dynamics and deviance‐related responses across developmental stages. Across developmental stages, a total of 35 recordings (7 cultures × 5 DIVs) were included in this analysis. Recording‐level comparisons were used to examine the relationship between avalanche statistics and DDIs across developmental stages.

The avalanche size distributions of cultures at different DIVs exhibited a gradual shift in their statistical properties, from predominantly exponential‐like profiles toward distributions with increasingly heavy‐tailed characteristics, including power‐law–like behavior (Figures 2 and 6A–C). At early developmental stages (e.g., DIV10), distributions were largely consistent with subcritical dynamics, whereas with maturation, an increasing proportion of cultures exhibited heavy‐tailed activity patterns.

**FIGURE 6:**
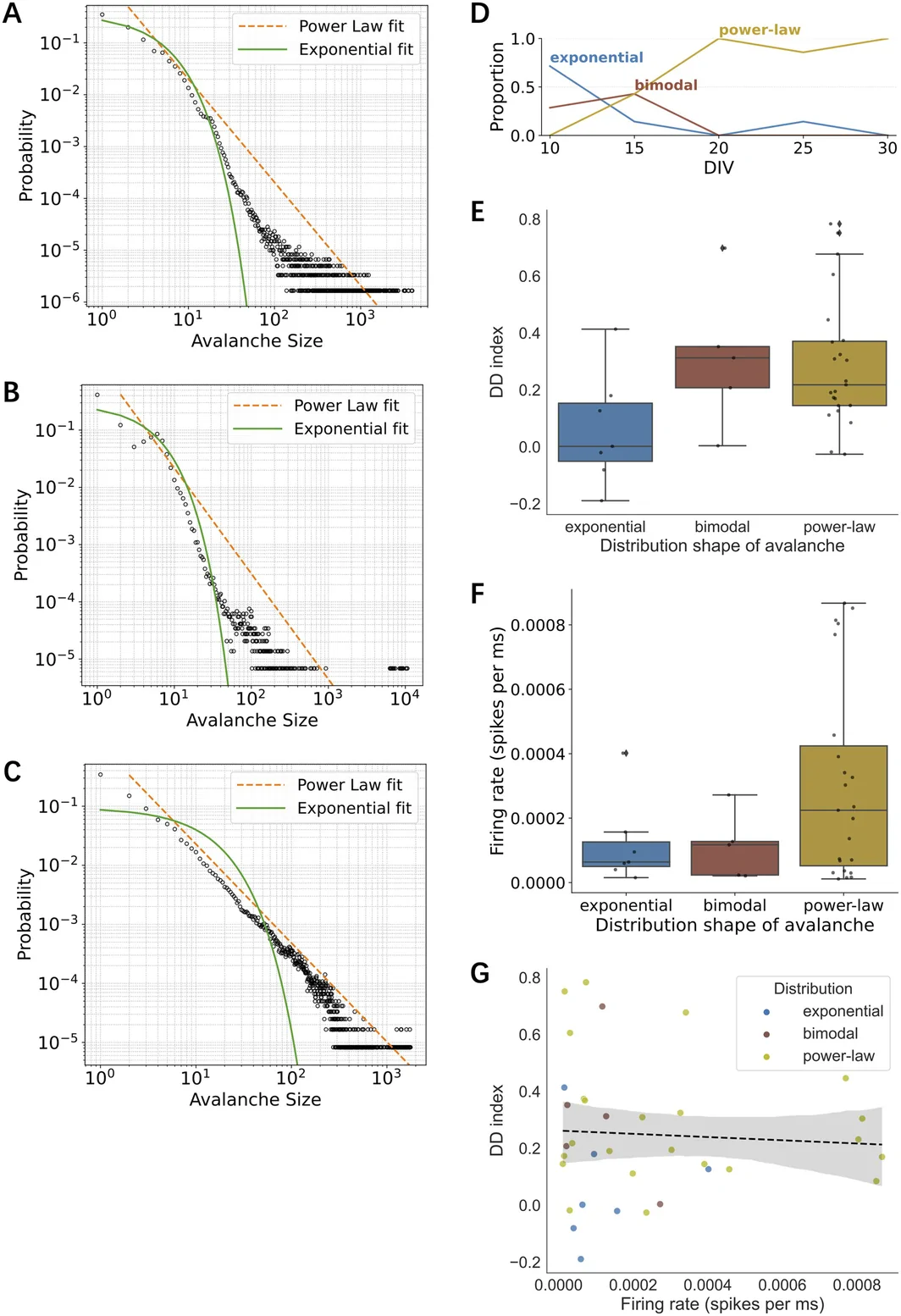
Relationship between avalanche dynamics and deviance detection. (A–C) Representative avalanche size distributions from cultures categorized as (A) exponential, (B) bimodal, and (C) power‐law types based on overall distributional features (see Section 2). The bimodal category reflects the presence of occasional large avalanches and does not imply strict statistical bimodality. (D) Proportion of cultures assigned to each avalanche category across developmental stages (DIV10–30). (E) Deviance detection index (DDI) across avalanche categories. (F) Spontaneous firing rates across avalanche categories. (G) Relationship between DDI and firing rate, assessed using Spearman correlation. Each data point represents one culture recorded at a given developmental stage (total observations = 35 from 7 independent cultures across DIV10–30). Recording‐level distributions are shown in (E–G). Inferential analysis of DDI across avalanche categories was based on the mixed‐effects model accounting for developmental stage and repeated measurements, as described in Sections 2 and 3.

Following the classification criteria described in the method, distributions were categorized into exponential, bimodal, or power‐law types. We note that this classification is operational and reflects overall distributional regimes rather than strict statistical model identification. In particular, the bimodal category reflects the presence of occasional large avalanches and does not imply strict statistical bimodality. Exponential‐type distributions predominated at DIV10, consistent with immature, subcritical dynamics (Pasquale et al. 2008; Yada et al. 2017), but became less prevalent with further development. Bimodal distributions were mainly observed at DIV10 and DIV15, whereas power‐law–type distributions first appeared around DIV15 and became more prevalent after DIV20 (Figure 6D). Importantly, the transition between avalanche regimes was gradual and heterogeneous across cultures, rather than characterized by sharp or discrete state switches, consistent with a continuous shift in network dynamics during development.

We next examined recording‐level differences in DD across these avalanche categories. DDI values were lowest in recordings classified as exponential and higher in those classified as bimodal or power‐law (Figure 6E). Recording‐level comparisons showed the clearest difference between exponential and power‐law recordings (Cliff's *δ* = −0.565).

For inferential testing while accounting for developmental stage and repeated measurements, we analyzed DDI using a mixed‐effects model with avalanche category and DIV as fixed effects and culture as a random intercept. The model showed a significant overall association between avalanche category and DDI (Wald *χ* ^2^(2) = 7.827, *p* = 0.0200). Pairwise contrasts indicated higher DDI for power‐law than exponential distributions (Holm‐adjusted *p* = 0.0247), whereas the bimodal category did not differ significantly from either exponential (*p* = 0.0856) or power‐law (*p* = 0.4181).

We next examined whether this relationship was accompanied by differences in overall network activity (Figure 6F). At the recording level, FRs showed no clear differences among avalanche categories, with small to negligible effect sizes (Cliff's *δ* range −0.36 to −0.03). The recording‐level association between DDI and spontaneous FR was also weak (Spearman *ρ* = −0.111; Figure 6G). The association between avalanche category and DDI remained significant in the mixed‐effects analysis accounting for developmental stage and repeated measurements, whereas no clear recording‐level association between DDI and spontaneous FR was observed.

Together, these findings indicate that DD is already present in cultures exhibiting bimodal avalanche statistics, suggesting that a fully developed power‐law regime is not required for its emergence. DDI was low in cultures exhibiting exponential activity, whereas differences between bimodal and power‐law regimes were less pronounced. These results suggest that critical dynamics are not required for DD and that DD is already present under near‐critical conditions, with the clearest difference observed between power‐law and exponential activity.

### 3.2 Influence of Early Structured Stimulation on Mature Responses

We evaluated whether early structured stimulation is associated with differences in deviance‐detection sensitivity at maturity. At DIV31–35, we compared two groups of cultured cortical networks: an experimental group that had undergone five structured stimulation sessions during the immature period (DIV10–30) and a control group without prior structured stimulation. Each group comprised six independent cultures, and all statistical analyses were performed at the culture level.

In both groups, neurons exhibited deviant responses under the oddball paradigm (Figure 7A,D). In these mature‐stage analyses (DIV31–35), the late response window was defined as 11–150 ms, consistent with previous studies in mature cultures (Zhang et al. 2025). Time‐resolved normalized response differences further indicated deviance sensitivity in both groups (Figure 7B,E), suggesting that deviance sensitivity was present at maturity regardless of prior stimulation experience. SSAI values for deviant stimuli were significantly greater than those for MSC stimuli in the control group (*p* = 0.031, two‐sided paired Wilcoxon signed‐rank test; Figure 7F), whereas this difference did not reach statistical significance in the experimental group (*p* = 0.063; Figure 7C). DDI values were significantly above zero in both groups (experimental: *p* = 0.031; control: *p* = 0.016; one‐sided Wilcoxon signed‐rank tests, alternative DDI > 0; Figure 7G), confirming the presence of DD in both groups. SSAI and DDI capture related but distinct aspects of the response, with DDI directly quantifying the DEV–MSC difference. SSAI values for deviant stimuli were significantly greater than zero in both groups (two‐sided one‐sample Wilcoxon signed‐rank test, *p* = 0.031 for both groups). SSAI values for MSC stimuli were not significantly different from zero in the experimental group (*p* = 0.094) but were significant in the control group (*p* = 0.031; two‐sided Wilcoxon signed‐rank test).

**FIGURE 7:**
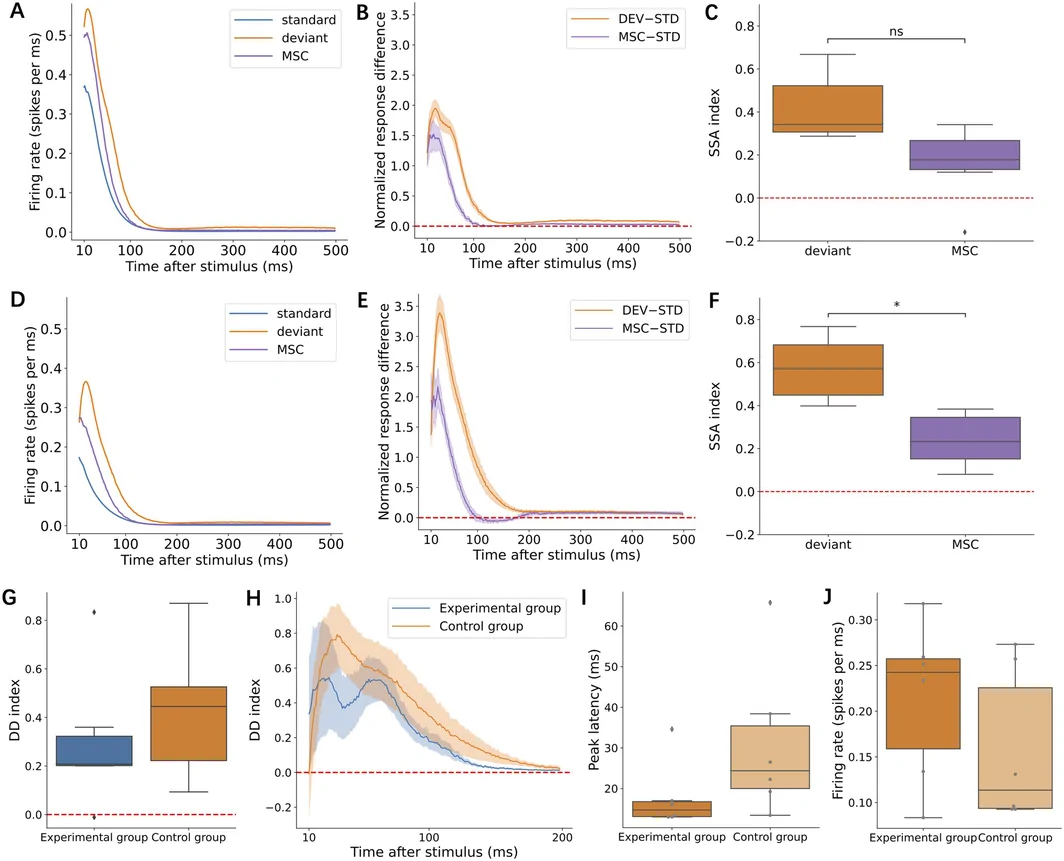
Effects of early structured stimulation on mature deviance detection. (A–C) Experimental group (*n* = 6 cultures). (A) Population‐averaged peri‐stimulus time histograms (PSTHs). (B) Time‐resolved normalized DEV–STD and MSC–STD response differences. (C) SSAI values comparing deviant and MSC responses, computed within the late response window (11–150 ms). Shaded areas in (A, B) indicate mean ± SEM across cultures. (D–F) Control group (*n* = 6 cultures). (D) Population‐averaged PSTHs. (E) Time‐resolved normalized DEV–STD and MSC–STD response differences. (F) SSAI values comparing deviant and MSC responses, computed within the late response window (11–150 ms). Shaded areas in (D, E) indicate mean ± SEM across cultures. (G–J) Culture‐level comparisons between experimental and control groups (*n* = 6 cultures per group). Each data point represents one independent culture. (G) Deviance detection index (DDI), computed within the late response window (11–150 ms). (H) Time‐resolved DDI (10–200 ms post‐stimulus; mean ± SEM across cultures). (I) Peak latency of deviant responses. (J) Mean DEV‐evoked firing rate during 0–150 ms after stimulation. Within‐group comparisons in (C) and (F) used Wilcoxon signed‐rank tests: *p* < 0.05 (test sidedness is given in Section 3). Between‐group comparisons used two‐sided Mann–Whitney *U* tests with Benjamini–Hochberg FDR correction across DDI, peak latency, firing rate, and response duration. Mature‐stage values were averaged across DIV31–35 within each culture before group comparisons.

Between‐group comparisons of mature responses used one value per culture, obtained by averaging DIV31–35 measurements (*n* = 6 cultures per group). The combined response profile—DDI, peak latency, DEV‐evoked FR, and response duration—did not differ significantly between groups (PERMANOVA on *z*‐standardized features; pseudo‐*F*(1,10) = 1.465, *R* ^2^ = 0.128, exact *p* = 0.258).

None of the four individual measures differed significantly after FDR correction (two‐sided Mann–Whitney *U* tests: DDI, *U* = 13.0, *p*_FDR = 0.485; peak latency, *U* = 6.5, *p*_FDR = 0.309; DEV‐evoked FR, *U* = 23.0, *p*_FDR = 0.485; response duration, *U* = 12.0, *p*_FDR = 0.485). Control cultures had descriptively higher DDI values (Figure 7G,H), whereas stimulated cultures had earlier peak responses (Figure 7I) and higher DEV‐evoked FRs (Figure 7J). The largest descriptive difference was in peak latency, but even its unadjusted comparison was not significant (raw *p* = 0.077). A sensitivity analysis retaining individual DIV31–35 recordings likewise found no significant group effect or group‐by‐DIV interaction after FDR correction. Response durations for standard, deviant, and MSC responses in the experimental and control groups are summarized descriptively in Table 2.

**TABLE 2:** Response duration in experimental and control groups at maturity. Response duration (ms, mean ± SD) for standard (STD), deviant (DEV), and many‐standards control (MSC) stimuli in experimental and control cultures during the mature stage (DIV31–35). Values were computed at the level of independent cultures (experimental group *N* = 6, control group *N* = 6).

Groups	STD	DEV	MSC
Experimental group (ms)	155.3 ± 39.0	139.7 ± 18.6	144.2 ± 49.6
Control group (ms)	173.8 ± 65.9	160.5 ± 33.3	118.2 ± 48.2

We also tested whether early stimulation was associated with a different trajectory of spontaneous network dynamics. Applying the Study 1 avalanche‐analysis pipeline to Study 2 recordings showed a shift from exponential and bimodal toward power‐law–like distributions in both groups during DIV10–30 (Figure 8A). By DIV30, all recordings in both groups were classified as power‐law–like. The overall developmental trajectories did not differ significantly (culture‐level exact permutation test, *p* = 0.561).

**FIGURE 8:**
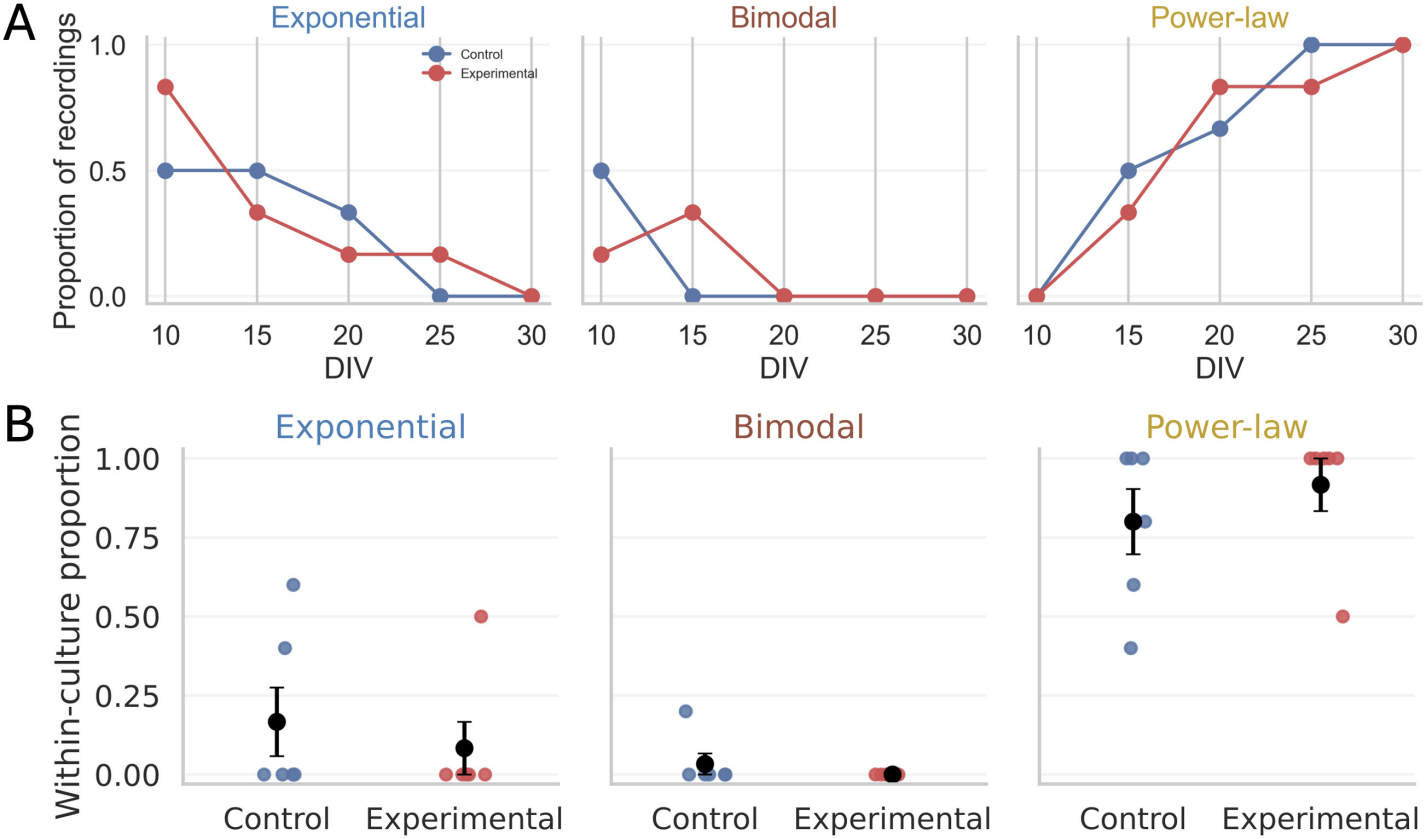
Avalanche‐category trajectories and mature‐stage composition in Study 2. (A) Proportion of recordings classified as exponential, bimodal, or power‐law–like at each DIV during DIV10–30, shown separately for control and experimental cultures. (B) Within‐culture proportions of DIV31–35 recordings in each category. Each colored point is one culture; black points and error bars show the mean ± SEM across cultures (*n* = 6 per group). Each culture contributes equally, irrespective of its number of available recordings.

At DIV31–35, we calculated each culture's proportions of recordings in the three avalanche categories and compared these proportions between groups (*n* = 6 per group; Figure 8B). Power‐law–like recordings predominated in both groups (control: 80.0%; experimental: 91.7%). Neither the overall category composition (*p* = 0.576) nor the proportion of power‐law–like recordings (*p* = 0.424) differed significantly.

At maturity, both groups exhibited DD and predominantly power‐law–like avalanche dynamics. The stimulated group showed earlier peak responses and lower DDI values than controls, although these differences were not statistically significant.

## 4 Discussion

This study systematically examined the developmental trajectory, network dynamics, and experience‐dependent modulation of DD in dissociated cortical networks. We found that DD strengthened during development: SSA was detectable from approximately DIV15, whereas robust differentiation between deviant and control responses was consistently observed by later stages, accompanied by faster and more temporally precise population activity. At the network level, avalanche analysis showed that cultures whose activity more closely approximated critical (power‐law statistics) tended to exhibit stronger DD sensitivity, whereas subcritical (exponential) cultures displayed weaker effects. Notably, deviance‐related responses were already detectable in cultures with bimodal avalanche distributions, indicating that critical dynamics are not strictly required for their occurrence. In Study 2, both groups shifted toward power‐law–like avalanche distributions; neither their developmental trajectories nor their mature evoked responses differed significantly. Together, these findings indicate that developing dissociated cortical networks can support deviance‐sensitive computations, with ongoing self‐organization of network dynamics and early experience potentially modulating their strength and temporal characteristics.

### 4.1 Self‐Organized Maturation of DD

Local cortical slices have been shown to learn temporal information through optical stimulation and to exhibit predictive capabilities independent of top‐down inputs (Liu and Buonomano 2025). Similarly, dissociated cortical neurons derived from rat embryos display sensitivity to both deviance and regularity, suggesting that prediction‐related response dynamics can emerge even in simplified cortical networks (Zhang et al. 2025). Our findings further support the view that DD can arise during maturation in dissociated cortical networks.

SSA emerged around DIV15 and became progressively more stable and efficient with further development, as indicated by increasingly differentiated responses to deviant versus standard stimuli and progressively shorter responses. This developmental trajectory differs from that observed in the rat auditory cortex (A1), where SSA matures gradually with a decrease in common SSAI (Valerio et al. 2024). DD, a process beyond simple adaptation, also appeared around DIV15 and remained detectable through DIV30.

These results indicate a sequential developmental progression in which adaptation to regular input and sensitivity to deviance emerge alongside ongoing network maturation. They are broadly consistent with the idea that self‐organizing network dynamics can support response properties relevant to predictive‐coding accounts, without directly demonstrating hierarchical predictive coding in this preparation. The developmental shortening of response durations and peak latencies of deviance further suggests that information processing becomes faster and more temporally efficient in mature networks, analogous to the decrease in MMR latency observed in vivo as neural circuits refine (Näätänen et al. 2019).

One potential concern when using electrical stimulation paradigms is that deviance‐related response differences could arise from partial spatial overlap between stimulation territories, leading to differential local adaptation. Our additional analysis confirmed substantial response overlap across stimulation sites (Figure 5). The analysis measured overlap of evoked spiking responses rather than adaptation itself, so recruitment below the response‐detection threshold and other effects of stimulation history remain possible. The slope analysis detected no significant increase in DEV–MSC with overlap load. The late DEV–MSC difference was nevertheless positive in recording channels with no detectable response to any other MSC stimulation site. This finding argues against measured cross‐site overlap as the sole explanation for the late DEV > MSC effect.

In addition, the observed differences between SSAI and DDI may reflect not only differences in metric definitions but also, in principle, variability in underlying adaptation patterns across conditions. Although STD, DEV, and MSC were compared using the same stimulation site, the stimuli preceding it differed between the oddball and MSC sequences. Matching the physical test site and its presentation probability therefore did not match the full stimulation history.

Several limitations of the present study should be acknowledged. First, because cultures in the developmental experiment received repeated stimulation across successive developmental stages, the observed developmental trajectory likely reflects both maturation and cumulative effects of prior stimulation and therefore does not fully isolate stimulation‐naïve emergence of DD. The oddball and MSC paradigms thus served both as probes of DD and as potential developmental perturbations. Study 2 showed that a shift toward power‐law–like avalanche distributions also occurred without early structured stimulation. This result concerns spontaneous network dynamics; it does not isolate stimulation‐naïve development of DD.

In addition, because the MSC condition was typically presented after the oddball runs, order‐related effects such as response fatigue could, in principle, influence the observed differences. However, several observations argue against a dominant role of such effects. In our previous work (Zhang et al. 2025), neural responses recovered on a timescale of seconds to tens of seconds following stimulation, suggesting that the 5‐min rest intervals used here were sufficient to allow recovery of response amplitudes. Moreover, in the present study, comparisons of pre‐ and post‐stimulation spontaneous activity did not reveal clear changes in avalanche distributions or delta *p*, arguing against a substantial shift in global network dynamics induced by the stimulation sequence.

Furthermore, all experiments were performed in dissociated primary cortical cultures, which lack the laminar organization, long‐range inputs, and developmental constraints characteristic of intact cortical circuits. As a result, the network mechanisms giving rise to the deviance‐related responses observed here may differ from those operating in vivo. Importantly, rather than establishing a general or exclusive mechanism, our findings delineate the minimal network conditions under which deviance‐related responses can be observed in vitro. In this sense, dissociated cultures provide a tractable experimental platform for isolating how local network dynamics, developmental state, and stimulation experience shape responses to unexpected inputs, without implying a one‐to‐one correspondence with mechanisms in the mature brain.

In summary, our findings demonstrate that DD and SSA can be supported in dissociated cortical networks without requiring explicit hierarchical circuit organization, emerging alongside self‐organizing network dynamics. These results establish dissociated cultures as an experimentally tractable model for studying predictive computation (Mill et al. 2011; Kern and Chao 2023).

### 4.2 Critical Dynamics as a Substrate for DD

Numerous studies have shown that dissociated neural networks gradually transition from subcritical to critical dynamics during development (Kamioka et al. 1996; Pasquale et al. 2008; Sun et al. 2010; Tetzlaff et al. 2010; Yada et al. 2017). Consistent with this, we found that early in vitro activity exhibited exponentially distributed avalanche sizes, indicative of a subcritical regime. As synaptic connectivity matured, cascades grew larger and more frequent, yielding bimodal avalanche size distributions characterized by many small events interspersed with occasional large bursts. These may reflect the predominance of excitatory synapses during early culture stages (Chiappalone et al. 2006). By DIV20–30, mature cultures displayed power‐law avalanche size distributions—a hallmark of self‐organized criticality (SOC), in which event sizes span a broad range without a characteristic scale. As development progressed, inhibitory synapses strengthened, possibly through GABA‐mediated mechanisms, allowing networks to self‐organize toward a balanced state (Baho and Di Cristo 2012; Le Magueresse and Monyer 2013; Plenz et al. 2021). This shift fragmented large bursts into more controlled propagation (Yada et al. 2017).

Notably, cultures approaching a power‐law distribution exhibited the strongest DD, whereas subcritical cultures (exponential distributions) showed markedly weaker deviance sensitivity, with intermediate (bimodal) networks displaying moderate DD sensitivity. These results suggest that near‐critical dynamics may provide a favorable regime for deviance‐sensitive processing in this preparation. Importantly, no clear association between DDI and spontaneous FR was detected at the recording level. Thus, the association between avalanche category and DDI was not accompanied by a corresponding relationship with spontaneous FR.

This observation aligns with theoretical predictions that neural systems operating near criticality maximize dynamic range and information transmission, thereby optimizing the propagation and integration of prediction‐error signals (Beggs and Plenz 2003; Shew et al. 2009, 2011; Shew and Plenz 2013; Wilting and Priesemann 2019). In such networks, small perturbations can propagate widely without destabilizing the system, enabling flexible yet stable encoding of statistical regularities and rapid updating of internal models when deviations occur. These theoretical accounts do not establish a causal direction in our data. Near‐critical dynamics may facilitate DD, repeated stimulation may alter network dynamics, or both may develop together during maturation. In Study 2, the shift toward power‐law–like avalanche distributions occurred in cultures both with and without early structured stimulation, and no significant group difference was detected in the developmental trajectory or mature category composition.

### 4.3 Synaptic Mechanisms Underlying DD

At the synaptic and circuit level, deviance‐related responses in cultured cortical networks are likely shaped by coordinated excitatory and inhibitory interactions. Previous work has shown that selective adaptation and enhanced responses to rare inputs can arise naturally in developing cortical cultures through the interplay of excitatory synaptic depression and fast GABAergic transmission, which together implement network‐level gain control (Eytan et al. 2003). In parallel, NMDA receptor–dependent late‐phase MMRs have been demonstrated in dissociated cortical cultures (Zhang et al. 2025), suggesting that slower recurrent synaptic dynamics and plasticity mechanisms contribute to the amplification and stabilization of deviance sensitivity.

Importantly, excitation–inhibition balance is closely linked to the emergence of critical network dynamics, a relationship that has been highlighted in recent work on spiking neural networks (Ikeda et al. 2025) and represents a central feature of the present study. During development, the gradual maturation of inhibitory synapses is expected to shift network activity from subcritical toward near‐critical regimes. In such regimes, recurrent dynamics can sustain longer fading memory of past inputs, thereby enhancing the network's ability to integrate stimulus history. This property may provide a mechanistic basis for the observed increase in deviance‐detection sensitivity (DDI) during development, as stronger temporal integration allows more effective comparison between expected and incoming stimuli.

These considerations suggest that deviance‐related responses are unlikely to reflect purely excitatory mechanisms but instead emerge from coordinated excitation–inhibition dynamics, whose developmental tuning shapes both network criticality and the temporal integration processes underlying DD. Future studies that directly manipulate inhibitory transmission or excitation–inhibition balance will be important for establishing the causal contribution of these mechanisms to deviance sensitivity in cultured networks.

These network‐level interpretations are broadly consistent with known timelines of synaptic maturation in dissociated cortical cultures. In vitro studies of synapse development show that synapse formation is most pronounced during the first 15 days and remains stable up to DIV60. The NMDA receptor subunit NR1 and AMPA receptor subunits GluR2/GluR3 are already expressed by DIV5, preceding the appearance of synaptic markers, and glutamate receptor levels peak by DIV10–15 before stabilizing (Lesuisse and Martin 2002). Similarly, between DIV7 and DIV14, large increases in the amplitudes and frequencies of spontaneous excitatory postsynaptic currents (EPSCs) have been observed in E18 cultures. EPSCs are primarily mediated by AMPA receptors at DIV7 but involve both NMDA and AMPA receptors at DIV14 (Lin et al. 2002). Moreover, in cortical neurons, the total number of excitatory synapses increases significantly only after DIV21, continuing through DIV28, whereas inhibitory synapses increase significantly only after DIV28 (Harrill et al. 2015).

The developmental trajectory of DD observed in the present cultures appears to parallel previously reported timelines of synaptic maturation involving NMDA receptor–mediated transmission. Therefore, we speculate that at DIV10, responses were largely confined to fast components, likely reflecting predominantly AMPA‐mediated activation. By DIV15, increasing NMDA receptor involvement may have contributed to the emergence of prominent late responses, and after DIV15, DD sensitivity became more consistently expressed in parallel with the gradual maturation and stabilization of synaptic connectivity.

### 4.4 Experience‐Dependent Tuning of Temporal Dynamics

DD was evident in cultures with and without early structured stimulation at maturity (DIV31–35). Stimulated cultures exhibited earlier peak responses and slightly lower DDI values than controls, although these group differences were not statistically significant. If a stimulation effect is confirmed in future experiments, candidate mechanisms include NMDA receptor–mediated long‐term potentiation (LTP) and spike‐timing–dependent plasticity (STDP). NMDA receptor–mediated LTP strengthens synaptic efficacy (Bliss and Collingridge 1993), whereas STDP refines connectivity by reinforcing causally effective, shorter latency pathways (Bi and Poo 1998; Song et al. 2000), thereby potentially reducing response latency without necessarily enhancing response contrast. Within predictive processing frameworks, NMDA‐dependent plasticity has been proposed to contribute to the refinement of temporal prediction mechanisms (Friston 2005; Garrido et al. 2009).

Supporting this interpretation, recent in vitro studies demonstrate that repeated stimulation can reorganize excitatory–inhibitory pathways, enhancing pattern recognition (Shao et al. 2025; Ikeda et al. 2025) and enabling spontaneous replay of learned temporal sequences (Liu and Buonomano 2025). In vivo, passive sensory exposure can induce long‐term memory traces even in the adult auditory cortex when stimuli are sufficiently complex (Kurkela et al. 2016), whereas inappropriate early exposure (e.g., noise rearing) disrupts the normal development of SSA and deviance sensitivity (Wang et al. 2019). Similarly, Valerio et al. (2024) showed that both deprivation and overexposure during critical periods distort the maturation of SSA, underscoring the bidirectional plasticity of experience‐dependent sensory processing mechanisms. Together, these findings support the possibility that early structured stimulation may produce lasting changes in functional circuitry that can influence subsequent deviance‐related response dynamics.

From a broader perspective, our results highlight how early sensory experience may influence the temporal dynamics and differentiation of deviance‐related responses. The descriptive patterns in response timing and deviance sensitivity identify specific features for examining the contribution of early experience. Altered MMN amplitude and latency have been reported in adolescents with neurodevelopmental disorders such as autism and ADHD (attention deficit hyperactivity disorder) (Chen et al. 2020; Yamamuro et al. 2016; Di Lorenzo et al. 2020; Lassen et al. 2022; Schwartz et al. 2018). While the present findings do not address the mechanisms underlying these alterations, they illustrate how early activity patterns may contribute to variability in the temporal and response‐selective properties of developing networks. Beyond basic neuroscience, controlled stimulation paradigms may therefore provide a useful framework for investigating how early activity patterns influence the development of biological and artificial neural systems.

During the developmental period (DIV10–30), network activity undergoes progressive maturation, shifting from subcritical toward near‐critical dynamics. This transition is associated with stronger and more differentiated deviance‐related responses, linking the emergence and persistence of DD to progressive network reorganization. While prior stimulation may contribute through synaptic plasticity, the present design does not separate its contribution from that of maturation. In this self‐organized maturation, response strength, temporal precision, and differentiation between deviant and control conditions all increase.

To examine whether the descriptive group differences in evoked responses were accompanied by broader differences in network state, we compared mature avalanche dynamics. Power‐law–like recordings predominated in both groups, with no significant difference in category composition (Figure 8B). Thus, the descriptive differences in evoked responses were not accompanied by a clear shift toward a more non‐critical avalanche regime in the stimulated group. This classification did not measure fatigue directly, and the small number of independent cultures limited the precision of group estimates.

Together, these findings show that cortical cultures with and without early structured stimulation both express DD at maturity, alongside predominantly power‐law–like network dynamics.

## 5 Conclusion

Our findings show that DD in dissociated cortical cultures emerges around DIV15 and remains detectable through DIV30, as stimulus‐evoked responses become stronger, faster, and more temporally precise—consistent with the progressive establishment of recurrent synaptic interactions. At the network level, cultures that exhibited power‐law avalanche statistics showed higher DDI than those with exponential distributions. Importantly, DD was already present in cultures with bimodal avalanche distributions and was not significantly different from power‐law cultures in our sample, indicating that fully developed critical dynamics (power‐law avalanche statistics) are not required for the emergence of DD. Rather, network states approaching criticality were associated with stronger deviance‐related processing as networks matured. Cultures with and without early stimulation both shifted toward power‐law–like avalanche distributions. At maturity, DD was detectable in both groups, and power‐law–like avalanche dynamics predominated. The observed group differences in response timing and DDI were not statistically significant. This reduced preparation therefore provides a useful framework for studying local circuit and network factors associated with deviance‐related responses, with potential implications for biologically inspired adaptive computation.

## Author Contributions

**Zhuo D. Zhang:** conceptualization, data curation, formal analysis, investigation, visualization, methodology, software, validation, writing – original draft. **Amit Yaron:** methodology, project administration, supervision, validation, writing – review and editing. **Dai Akita:** funding acquisition, methodology, project administration, resources, supervision, validation, writing – review and editing. **Tomoyo Isoguchi Shiramatsu:** funding acquisition, project administration, supervision, writing – review and editing. **Zenas C. Chao:** validation, writing – review and editing. **Hirokazu Takahashi:** conceptualization, project administration, resources, supervision, funding acquisition, writing – review and editing, validation.

## Ethics Statement

All animal experimental protocols were approved by the Graduate School of Information Science and Technology's Ethics Committee at the University of Tokyo (JA21‐8). All experimental procedures were carried out in accordance with these approved guidelines.

## Conflicts of Interest

The authors declare no conflicts of interest.

## Data Availability

The processed data and analysis code supporting the findings of this study are publicly available in Zenodo at https://doi.org/10.5281/zenodo.19388937. Additional data are available from the corresponding author upon reasonable request.
